# Dementia: changes from ICD-10 to ICD-11

**DOI:** 10.1007/s00115-025-01885-9

**Published:** 2025-10-14

**Authors:** Frank Jessen, Karl Broich

**Affiliations:** 1 Department of Psychiatry, Faculty of Medicine, University Hospital Cologne, Kerpener Straße 62, 50937 Cologne, Germany; 2 German Center for Neurodegenerative Diseases (DZNE), Cologne/Bonn, Germany; 3 Federal Institute for Drugs and Medical Devices (BfArM), Bonn, Germany

**Keywords:** Severity level, Postcoordination, Alzheimer’s disease, Mild neurocognitive disorder, Biomarkers, Schweregrade, Postkoordination, Alzheimer-Krankheit, Leichte neurokognitive Störung, Biomarker

## Abstract

The International Statistical Classification of Diseases and Related Health Problems version 11 (ICD-11) represents a conceptual advance over ICD-10 in the classification of dementias. Although the syndromic classification in the chapter “Neurocognitive disorders” remains in principle unchanged, the introduction of severity levels and the central positioning of mental and behavioral symptoms enables a more precise coding of the clinical diagnoses. Furthermore, the introduction of mild neurocognitive disorder as a prodromal state of dementia is new. The clinical criteria developed by international experts, e.g., for frontotemporal dementia or Lewy body disease, are not yet sufficiently included in ICD-11. Biomarkers for the etiological diagnostics of dementia are also not mentioned, so that it is unclear which role they play in the disease classification in ICD-11. Due to the rapid development in the field of neurodegenerative diseases, regular updates would be desirable.

## Abstract (de)

Die International Statistical Classification of Diseases and Related Health Problems 11 (ICD-11) stellt einen konzeptuellen Fortschritt zur ICD-10 bei der Klassifikation von Demenzen dar. Die syndromale Einteilung im Kapitel Neurokognitive Störungen bleibt zwar prinzipiell erhalten, die Einführung von Schweregraden und die zentrale Codierung von psychischen und Verhaltensstörungen erlauben aber eine präzisere Erfassung der klinischen Diagnosen. Neu ist ferner die leichte neurokognitive Störung als Frühstadium von Demenzerkrankungen. Noch nicht ausreichend aufgenommen wurden die von internationalen Experten erarbeiteten klinischen Kriterien wie z. B. für die frontotemporale Demenz oder die Lewy-Körper-Krankheit. Auch Biomarker für die ätiologische Diagnostik von Demenzen werden nicht aufgeführt, sodass unklar bleibt, welche Rolle sie bei der Krankheitszuordnung in ICD-11 spielen. Aufgrund der schnellen Entwicklung im Feld neurodegenerativer Erkrankungen wären regelmäßige Aktualisierungen wünschenswert.

## Background

A conceptual shift is currently taking place in the field of dementia. Until recently, dementias were diagnosed as syndromes corresponding to etiological entities. Clinical criteria were defined for all common forms of dementia (e.g., Lewy body dementia, frontotemporal dementia; [7, 8]). In recent years biomarkers for the pathology have been added, particularly for Alzheimer disease in terms of markers for amyloid and tau aggregation [6], enabling a reliable etiological diagnosis. Biomarkers are also being developed for other forms of dementia. The possibility of biomarker-based detection of Alzheimer disease that is already at the mild cognitive impairment (MCI) stage means that, according to current diagnostic criteria, Alzheimer disease can be diagnosed in cases of mild symptoms only that do not yet meet the criteria for dementia [4, 9]. The shift from clinical–syndromal to biomarker-based etiological diagnoses, including prodromal stages, will also take place in other types of dementia.

## ICD-10

Following the syndromal classification, various forms of dementia are listed in Chap. F0 of the ICD-10 (Table 1). Dementia in Alzheimer disease (F00.*) is divided into early onset under 65 years of age (F00.0) and late onset from 65 years of age (F00.1). Furthermore, the atypical or mixed form (F00.2) and dementia in Alzheimer disease, unspecified (F00.9) can be coded. In vascular dementia (F01), a distinction is made between acute onset (F01.0), multi-infarct dementia (F01.1), subcortical vascular dementia (F01.2), mixed cortical and subcortical dementia (F01.3), and other vascular (F01.8) and unspecified vascular dementia (F01.9). F02 lists dementias in diseases classified elsewhere. These include Pick disease (F02.0), Creutzfeldt–Jakob disease (F02.1), Huntington disease (F02.2), primary Parkinson syndrome (F02.3), HIV disease (F02.4), and other diseases classified elsewhere (F02.8), e.g., Lewy body disease (see below). F03 is used to code unspecified dementias and F04 is used to code organic amnestic syndrome not caused by alcohol or other psychotropic substances.

**Table 1:** Coding of primary dementia disorders and mild cognitive impairment in ICD-10 and ICD-11

ICD-10	ICD-11
*F00-F09: Organic, including symptomatic, mental disorders*	*06: Neurocognitive disorders*
F00: Dementia in Alzheimer disease	6D80: Dementia due to Alzheimer disease
F00.0: with early onset before the age of 65	6D80.0: Dementia due to Alzheimer disease with early onset
F00.1: with late onset	6D80.1: Dementia due to Alzheimer disease with late onset
F00.2: atypical or mixed type	6D80.2: Alzheimer disease dementia, mixed type, with cerebrovascular disease 6D80.3: Alzheimer disease dementia, mixed type, with other nonvascular etiologies
F00.9: unspecified	6D80.Z: Alzheimer disease dementia, onset unknown or unspecified
F01: Vascular dementia	6D81: Dementia due to cerebrovascular disease
F01.0: Vascular dementia of acute onset	Specification of 8 types of cerebrovascular diseases possible through post-coordination
F01.1: Multi-infarct dementia	–
F01.2: Subcortical vascular dementia	–
F01.3: Mixed cortical and subcortical vascular dementia	–
F01.8: Other vascular dementia	–
F01.9: Vascular dementia, unspecified	–
F02: Dementia in other diseases classified elsewhere	6D85: Dementia due to diseases classified elsewhere (10 diseases, see selection below)
F02.0: Dementia in Pick disease	6D83: Frontotemporal dementia (separate category)
F02.1: Dementia in Creutzfeldt–Jakob disease	6D85.5: Dementia due to prion disease
F02.2: Dementia in Huntington disease	6D85.:1 Dementia due to Huntington disease
F02.3: Dementia in Parkinson disease	6D85.0: Dementia due to Parkinson disease
F02.4: Dementia in HIV disease	6D85.3: Dementia due to HIV
F02.8: Dementia in other specified diseases classified elsewhere Lewy body(-ies) (disease) 14 additional diseases from various fields	6D85.Y: Dementia due to other specified diseases classified elsewhere 6D82: Dementia due to Lewy body disease (separate category)
F03: Unspecified dementia	6D8Z: Dementia, unknown or unspecified cause
F04: Organic amnesic syndrome, not induced by alcohol and other psychoactive substances	6D72: Amnestic disorder
F06: Other mental disorders due to brain damage and dysfunction and to physical disease	–
F06.7: Mild cognitive disorder	6D71: Mild neurocognitive disorder
U63.- Mental and behavioral disorders in dementia (7 syndromes)	6D86: Behavioral or psychological disturbances in dementia (7 syndromes)
*G30–G32: Other degenerative diseases of the nervous system (primary disease)*	*08: Disorders with neurocognitive impairment as a major feature*
G30: Alzheimer disease	8A20: Alzheimer disease
G30.0: Alzheimer disease with early onset	–
G30.1: Alzheimer disease with late onset	–
G30.8: Other Alzheimer disease	–
G30.9: Alzheimer disease, unspecified	–
G31.0: Circumscribed brain atrophy (frontotemporal dementia [FTD], Pick disease, progressive isolated aphasia)	8A21: Progressive focal atrophies 8A23: Frontotemporal lobar degeneration
A81.0: Dementia in Creutzfeldt–Jakob disease	–
G10: Dementia in Huntington disease	–
G20.: Dementia in Parkinson disease	–
B22: Dementia in human immunodeficiency virus (HIV) disease	–
G31.82: Lewy body(ies) (dementia) (disease)	8A22: Lewy body disease

Chap. F06 includes other mental disorders due to damage or dysfunction of the brain or a physical illness. Mild cognitive impairment (F06.7) can be coded here if an underlying brain disease or physical illness of another type can be identified. This includes MCI in the context of Alzheimer disease, although F06.7 is not primarily intended for disease causing dementia.

In the neurological chapter, Alzheimer disease is listed under G30. Other codable forms of dementia include frontotemporal dementia (G31.0), brain degeneration due to alcohol (G31.2), Huntington disease (G10), primary Parkinson syndrome (G20), and Lewy body disease (G31.82).

## ICD-11

ICD-11 incorporates current diagnostic classifications of neurodegenerative dementias (Table 1; [1]). In Chap. 6 (mental, behavioral, neurodevelopmental disorders), dementias are listed in the subchapter on neurocognitive disorders. The list includes dementia due to Alzheimer disease (6D80), divided into early onset before the age of 65 (6D80.0) and late onset after the age of 65 (6D80.1). Furthermore, mixed forms with vascular pathology (6D80.2) and with other neurodegenerative pathologies (6D80.3) are distinguished. The post-coordination function allows specific diseases to be assigned to the mixed forms.

Dementia caused by primary cerebrovascular disease (6D81) has a separate code. Here, various forms of cerebrovascular disease can be assigned with their own codes through post-coordination. Furthermore, the chapter lists dementia due to Lewy body disease (6D82); frontotemporal dementia (6D83); dementia due to psychoactive substances (6D84), including alcohol (6D84.0); dementia caused by sedatives, hypnotics, or anxiolytics (6D84.1); dementia caused by volatile inhalants (6D84.2) and by other unspecified psychoactive substances (6D84.3); as well as dementia caused by diseases classified elsewhere (6D85). For the latter code, nine diseases are listed, each of which can be linked via post-coordination. Remaining dementias are dementia due to diseases not classified elsewhere (D6586.Y) and dementia of unknown cause (D6586.Z).

All dementias can be classified as mild, moderate, or severe via post-coordination. Psychological and behavioral symptoms can be coded individually (psychotic symptoms 6D86.0, affective symptoms 6D86.1, anxiety symptoms 6D86.2, apathy 6D86.3, agitation or aggression 6D86.4, disinhibition 6D86.5, wandering 6D86.6, other 6D86.Y, unspecified 6D86.Z).

A new addition is mild neurocognitive disorder (6D71), which corresponds to the concept of MCI as an early symptomatic manifestation of a neurodegenerative disease prior to dementia. Through post-coordination, mild neurocognitive disorder can be linked to specific diseases, such as Alzheimer disease. Isolated amnestic disorder (6D72) must be distinguished from mild neurocognitive disorder.

In Chap. 8 (diseases of the nervous system), dementias are listed as diseases with neurocognitive impairments as their main feature. These include Alzheimer disease (8A20), progressive focal atrophies (8A21), including posterior cortical atrophy (8A21.0), other specified (8A21.Y) and unspecified (8A21.Z) progressive focal atrophies, Lewy body disease (8A22), frontotemporal lobar degeneration (8A23), and other specified (8A2Y) or unspecified (8A2Z) disorders. Post-coordination allows for the coding of severity of dementia for Alzheimer disease, Lewy body disease, and frontotemporal lobar degeneration. These diseases, however, cannot be combined with mild neurocognitive disorder.

Chapter 6 describes the codable entities with texts on symptoms and causes, providing a narrative understanding of the diseases and syndromes. Chapter 8 does not include these descriptions. Neither chapter mentions specific diagnostic criteria or biomarkers.

## Comments on ICD-11

In ICD-11, the classification of dementias and neurocognitive disorders has been largely adapted to the current state of knowledge [11]. The most common forms of dementia are specified [9].

## Revision of etiological categories and post-coordination

While retaining the syndromal classification of dementia types, terms have been updated in comparison with ICD-10. Differentiated coding options for mixed dementia types have been created, and terms that are no longer used (e.g., Pick disease) have been removed.

An important innovation is the cross-chapter post-coordination, which allows codes from other chapters to be used to describe clinical entities and syndromes as precisely as possible. This is evident, for example, in dementia due to cerebrovascular disease (6D81), where post-coordination with neurological Chap. 8 allows for an accurate etiological description of the underlying cerebrovascular disease.

However, like ICD-10, ICD-11 remains largely at the level of syndromes [11]. This is to be criticized in the case of Alzheimer disease, as etiological biomarkers based on cerebrospinal fluid and positron emission tomography (PET) are mature and in clinical use. Since syndromic diagnoses are considerably vague regarding the underlying pathology, it should be demanded that the etiology is defined by biomarkers. The development of blood-based biomarkers will improve the accessibility of these diagnostics [10]. In addition, the first therapies for Alzheimer disease are now available that require the determination of amyloid pathology with biomarkers [5]. Similar developments are also taking place in other neurodegenerative diseases, but are not yet as advanced.

Also, the clinical–syndromal classification is not closely aligned with the clinical criteria developed by international experts for individual dementia types, e.g., frontotemporal dementia including subtypes or Lewy body disease [7, 8]. Chapter 6 refers to these criteria in general terms, but not precisely. As a result, the specificity of clinical–syndromal diagnosis that could be achieved through the expert clinical criteria is not fully exploited, preventing the use of ICD-11 coding in clinical studies with new therapeutics.

There are also inadequate classifications that have been carried over from ICD-10. The division of Alzheimer disease into early-onset and late-onset forms, which is intended to distinguish between rare monogenic and frequent sporadic variants, cannot be maintained in this way. There are both monogenic cases of Alzheimer disease with onset after the age of 65 and non-monogenic cases with onset before that age [12]. There is no fundamental difference between the age groups in terms of diagnosis and treatment. An age-independent classification into monogenic and non-monogenic cases would be more useful. If the age classification is to be used for statistical purposes, it would have to be applied to all forms of dementia.

Another aspect is the lack of a syndromic description of atypical forms of Alzheimer disease. Logopenic aphasia, which is usually attributable to Alzheimer disease, is coded under frontotemporal dementias [2]. Posterior cortical atrophy, which is also predominantly caused by Alzheimer disease, can be coded in Chap. 8, but without reference to Alzheimer disease [3]. In both cases, a connection through post-coordination is not possible. Assuming that patients with these atypical forms of Alzheimer disease could benefit from anti-amyloid therapies, this classification is problematic.

## Healthcare-relevant coding

A compelling new feature in ICD-11 is the coding of dementia as mild, moderate, and severe, which is of major importance for healthcare planning and cost estimates. Another positive feature is that individual mental and behavioral symptoms can be coded for each dementia type through post-coordination. This is relevant for care planning, as mental and behavioral symptoms account for a large part of the medical and nursing resources required. In ICD-10, these symptoms can only be coded in Chap. U and are rarely used.

## Mild neurocognitive disorder

A key advancement in ICD-11 is the introduction of mild neurocognitive disorder—which was first introduced in the 5th edition of the *Diagnostic and Statistical Manual of Mental Disorders *(DSM-5)—with the possibility of assigning a specific etiology to this syndrome via post-coordination. This makes it possible to diagnose Alzheimer disease at the stage of mild neurocognitive disorder when, for example, the affected person can benefit from disease-modifying therapy. This diagnosis brings ICD-11 in line with research, which has a long history of focusing on the pre-dementia phase of neurodegenerative diseases in the development of diagnostics and therapy [5].

## Comparison of chaps. 6 and 8

The psychiatric chapter, Chap. 6, continues to use syndromes as key criteria (e.g., frontotemporal dementia), whereas the neurological chapter, Chap. 8, uses disease terms (e.g., Alzheimer disease) or morphological classifications (e.g., frontotemporal lobar degeneration) without reference to syndromal characteristics. The syndromal approach in Chap. 6 includes direct reference to disease severity and thus to the level of care required, but at the same time perpetuates the etiological uncertainty. This can be partially overcome through post-coordination. Chapter 8 corresponds to the etiological classification, but does not provide data that inform about disease stage-specific frequencies or treatment indications with the exception of those diagnoses that can be linked to mild, moderate, or severe dementia via post-coordination.

## Practical conclusion

- ICD-11 represents a clear improvement over ICD-10, particularly through the possibility to link syndromes with etiologies, to present care-relevant disease manifestations more centrally, and to diagnose mild neurocognitive disorder in the context of neurodegenerative diseases.
- With clinical syndrome-based diagnostics still in use, one limitation is that ICD-11 only loosely adheres to scientifically based clinical criteria for different dementias, and instead provides more general descriptions, which are associated with greater diagnostic inaccuracy and are therefore insufficient as inclusion criteria for clinical trials with new therapeutics.
- Biomarker-based etiological diagnostics of neurodegenerative diseases should be integrated into the further development of ICD-11.

## Declarations

This article does not contain any studies with human participants or animals performed by any of the authors.

The supplement containing this article is not sponsored by industry.
